# Positive sexuality, relationship satisfaction, and health: a network analysis

**DOI:** 10.3389/fpsyg.2024.1420148

**Published:** 2024-06-06

**Authors:** Giovanbattista Andreoli, Chiara Rafanelli, Paola Gremigni, Stefan G. Hofmann, Giulia Casu

**Affiliations:** ^1^Department of Psychology, University of Bologna, Bologna, Italy; ^2^Department of Psychology, Philipps-Universität Marburg, Marburg, Germany

**Keywords:** positive sexuality, relationship satisfaction, mental health, physical health, network analysis

## Abstract

**Introduction:**

Positive sexuality, defined as the happiness and fulfillment individuals derive from their sexual experiences, expressions, and behaviors, has been linked to relationship satisfaction and health. However, the intricate associations between positive sexuality and relationship functioning and health indicators have rarely been explored from a network perspective. This approach, by analyzing the interconnections among these factors within a broader system, can offer insights into complex dynamics and identify key variables for targeted interventions.

**Methods:**

The present study applied network analysis to uncover interconnections between positive sexuality, relationship satisfaction, and health indicators, highlight the most relevant variables and explore potential gender-based differences in a sample of 992 partnered individuals (51% women, aged 18–71 years). Networks were estimated via Gaussian Graphical Models, and network comparison test was used to compare men and women.

**Results:**

Results indicated that variables related to positive sexuality were more highly interconnected than the rest of the network. There were small-to-negligible connections between positive sexuality and relationship satisfaction variables, both of which had negligible or no connections with health. The network was globally invariant across gender, though a few connections were gender-specific. The most important variables, regardless of gender, related to pleasurable feelings during sexual intercourse.

**Discussion:**

The findings underscore the importance of enhancing positive sexual experiences within intimate relationships and have implications for research and clinical practice in positive sexuality.

## 1 Introduction

Traditionally, the biomedical model has approached sexual health from a problem-centered orientation, considering it as the absence of disease or disability (Masters and Johnson, 1970). Within a paradigm shift advocating for a more holistic view of sexual health and functioning, World Health Organization (2006) conceptualizes sexual health as a comprehensive state of well-being encompassing the psychological, physical, and social dimensions of sexual life. This conceptualization emphasizes the importance of a positive and respectful approach to sexuality and sexual relationships, ensuring sexual experiences that are pleasurable and secure. Within this sex-positive framework, positive sexuality is a novel construct defined as the fulfillent and contentment people experience with their sexual expressions and behaviors that contributes to their well-being (Williams et al., 2015). Positive sexuality and sexual satisfaction, although interconnected, encompass different aspects of sexual well-being. Positive sexuality encompasses a broader, more holistic view of sexuality that includes aspects like emotional intimacy, physical pleasure, positive sexual self-concept and experiences, and the potential for personal growth through sexual experiences (Williams et al., 2015, 2020). Sexual satisfaction more specifically entails a cognitive judgmental process in which the positive and negative aspects of one’s sexual relationships and the extent to which sexual experiences are rewarding are subjectively evaluated (Lawrance and Byers, 1995; Sánchez-Fuentes et al., 2014). Therefore, sexual satisfaction is one of the many components that make up the broader construct of positive sexuality. However, sexual satisfaction has been the most extensively studied positive indicator of sexuality to date (Syme et al., 2013; Sánchez-Fuentes et al., 2014), with the sex positive perspective gaining popularity only in recent years (Nimbi et al., 2021). Positive sexuality dimensions, including sexual satisfaction, have been linked to both personal and relational variables, including better physical and mental health and higher relationship satisfaction, respectively (Anderson, 2013; Sánchez-Fuentes et al., 2014; Sánchez-Fuentes and Sierra, 2015). Indeed, individuals with better physical health and healthier lifestyles report higher sexual satisfaction, sexual pleasure, and sexual desire (Flynn et al., 2016; Mollaioli et al., 2020; Gianotten et al., 2021). Furthermore, positive sexuality indicators such as sexual satisfaction and sexual wellbeing are positively associated with psychological well-being and self-esteem (Casu, 2019; Leavitt et al., 2019; Shaughnessy et al., 2022).

Relationship satisfaction is an important aspect of relationship quality that reflects an individual’s subjective evaluation of their partnership. This includes the extent to which their expectations and needs are met and their level of contentment with various aspect such as relational intimacy, communication, conflict resolution, and shared values and roles (Rusbult and Buunk, 1993; Graham et al., 2011; Topkaya et al., 2023). In support of the strong intertwining between sexual and relationship well-being, longitudinal findings reveal a bidirectional relationship between relational intimacy and sexual satisfaction (Beaulieu et al., 2023). Also, interventions that address sexuality in couples can enhance the quality of the relationship and vice versa. For example, interventions targeting sexual well-being in couples have been shown to improve not only aspects of positive sexuality such as fun, pleasure and comfort during sexual intercourse, but also emotional closeness and communication within the couple and relationship satisfaction (Rosier and Tyler, 2017; De Santis et al., 2019). Similarly, couple-based programs addressing issues related to emotional closeness and communication barriers can result in significant improvements in sexual intimacy and satisfaction (Wiebe et al., 2018; Morgis et al., 2019; Javadivala et al., 2021).

There is consistent evidence that relationship satisfaction is positively associated with various indicators of physical and mental health. For example, individuals who report high satisfaction with their relationships tend to exhibit lower stress levels and enhanced immune system functioning, which ultimately contributes to better physical health (Farrell and Simpson, 2017; Smith and Weihs, 2019; Shrout et al., 2020). Also, a higher relationship satisfaction has been linked to greater subjective well-being and lower psychological distress (Pieh et al., 2020; Mónaco et al., 2021; Tepeli Temiz and Elsharnouby, 2022), with some longitudinal studies reporting bidirectional effects (Gustavson et al., 2016; Roberson et al., 2018; Sağkal and Özdemir, 2020; Knobloch and Whisman, 2023).

Associations between positive sexuality, relationship functioning, and/or health indicators have rarely been explored from a network perspective. The network perspective conceptualizes psychological phenomena as dynamic systems of interrelated, interacting variables (Borsboom et al., 2021). Network analysis allows for the estimation of models comprising variables such as feelings, symptoms, or behaviors (i.e., nodes) and their interconnections (i.e., edges) (Hofmann et al., 2016). Centrality indices in network analysis can be used to gain insight into the most central or important nodes in a network, potentially enabling researchers and practitioners to identify key variables for targeted interventions (Opsahl et al., 2010).

One study that applied network analysis to identify psychological distress patterns in infertile patients found that the strongest network connection for both men and women was between sexual and relationship concerns (Cao et al., 2022). Another study of patients with post-traumatic stress disorder following childhood sexual abuse reported positive connections between sexual problems and psychiatric symptoms (Kratzer et al., 2020). Other evidence based on network analysis highlights a positive association of relationship satisfaction in terms of intimacy with overall quality of life among college students (Kuczynski et al., 2020). Some studies have reported gender differences in the associations among positive sexuality, relationship satisfaction, and physical and mental health. The positive effect of relationship satisfaction on sexual satisfaction was found to be stronger for women than for men (Kim and Jeon, 2013; Rausch and Rettenberger, 2021). Conversely, sexual satisfaction was a stronger predictor of subsequent relationship satisfaction for men than for women (Fallis et al., 2016; Cao et al., 2019). Aspects of sex related to physical pleasure showed stronger associations with mental health in men than in women (Stephenson et al., 2021; Jung et al., 2023), whereas the relational aspects of sexual intercourse were more important for women’s than men’s health (Omar et al., 2021; McKeen et al., 2022). Furthermore, relationship satisfaction was a stronger predictor of better mental health among women, while the reverse association of mental health with relationship satisfaction was reported to be more relevant for men (Whitton and Kuryluk, 2012; Roberson et al., 2018; Downward et al., 2022). However, other findings indicate no gender differences in the associations between positive sexuality indicators and relational satisfaction (Heiman et al., 2011; Schoenfeld et al., 2017; Roels and Janssen, 2020; Stephenson et al., 2021), between sexual satisfaction and subjective well-being or general health status (Buczak-Stec et al., 2019; Carcedo et al., 2020; Wang et al., 2023), and between relationship satisfaction and well-being (Gustavson et al., 2016; Morgan et al., 2018; Pollard and Rogge, 2022).

Using a network analysis approach, one study found that positive sexuality indicators such as sexual satisfaction, sexual pleasure, and sexual communication were associated with relationship satisfaction in the same way among men and women; however, sexual satisfaction was the most central variable in the network for men, while sexual desire was the most central variable for women (Nickull et al., 2022). Another study reported a positive edge between subjective health and relationship satisfaction in women only, and a negative edge between subjective health and sexual difficulties that was stronger in men than in women (Gocieková et al., 2024).

To the best of our knowledge, no study has investigated the interconnections between indicators of positive sexuality, relationship satisfaction, and health from a network perspective. The network approach has the advantage of providing a comprehensive understanding of a phenomenon by examining the relationships among all pairs of its components and identifying those that are more centrally positioned in the network (Borsboom et al., 2021). Therefore, the present study aimed to apply network analysis to examine the associations between positive sexuality, relationship satisfaction, and physical and mental health in a sample of partnered men and women, and to identify the most important (central) nodes in the network. In light of the inconsistent findings of previous studies, we also explored gender differences in the relationships among variables and in the centrality of nodes.

## 2 Materials and methods

### 2.1 Participants and procedure

This study used a cross-sectional design and was conducted in Italy. Recruitment was carried out using an exponential, non-discriminative snowball sampling method. This cost-effective approach involves participants recruiting future participants from among their acquaintances, ensuring a rapid growth of the sample without intentional selection based on gender or other demographic characteristics (Etikan et al., 2016). Specifically, individuals within the personal networks of the researchers received email and private messages containing a link to an anonymous online survey, and were requested to extend the invitation to their acquaintances. Inclusion criteria were being 18 years or older and having been in a stable heterosexual relationship for at least 1 year. The lower age limit of 18 was established to ensure all participants were of legal adult age, capable of giving informed consent independently. Conversely, no upper age limit was set to include a broad spectrum of adult experiences related to personal and relational functioning, to enhance the diversity of the sample. The initial page of the online survey presented a description of the study objectives, the inclusion criteria, the voluntary nature of participation, and the anonymity of the responses. To start the survey, the participants had to acknowledge their consent by checking a box. This study was approved by the Bioethics Committee of the University of Bologna (Prot. 0071562, 29 March 2019) and was conducted in accordance with the ethical standards for research involving human participants.

### 2.2 Measures

The online survey included a first section collecting information on sex, age, education, job status, cohabitation status, presence of children, and relationship length.

Positive sexuality was measured using the 5-item Positive Sexuality Scale (PSS) (Casu, 2019). Respondents were asked to think about their romantic relationship and assess the extent to which each item was representative of their sexual experience with their partner, using a 7-point scale ranging from 1 (“strongly disagree”) to 7 (“strongly agree”). The PSS has a one-factor structure that showed to be invariant across gender, age and fertility/childlessness status, with adequate reliability and positive associations with psychological well-being (Casu, 2019; Casu et al., 2021). Internal consistency in the present study was α = 0.95.

Relationship satisfaction was assessed with the marital satisfaction subscale of the ENRICH Marital Satisfaction Scale (EMS) (Fowers and Olson, 1993), which includes 10 items rated on a 5-point scale (1 = “strongly disagree” to 5 = “strongly agree”). This subscale showed adequate reliability and expected associations with relevant criterion variables (e.g., positive, large correlations with other measures of marital satisfaction) (Fowers and Olson, 1993). For the present study, we adapted items by replacing “marriage” with “relationship,” slightly modified the wording of one item (i.e., “I feel very good about how we each practice our religious beliefs and values” was changed to “I feel very good about how we each practice our beliefs and values”) and excluded one item referring to parenting status (“I am not satisfied with the way we handle our responsibilities as parents”). Therefore, 9 items were used in this study, with adequate internal consistency (α = 0.80).

Two single items were used for self-rated physical and mental health (Casu and Gremigni, 2019). Both items were rated on a 10-point scale from 1 (“poor”) to 10 (“excellent”). Single items are increasingly used in research as they demonstrated measurement soundness and usefulness as screening tools for the assessment of global physical and mental health (Östberg and Nordin, 2022; Galambos et al., 2023).

### 2.3 Data analysis

A network was built with individual items serving as nodes, where the connections between any two items were denoted by edges. We first selected items to be included as nodes in the network using the goldbricker approach (Jones, 2024). The goldbricker algorithm identifies nodes with highly similar patterns of connections with other nodes, indicating topological content overlap. It systematically compares the correlations of all possible node pairs with all other nodes in the network. Two nodes are considered to have topological content overlap if they differ only in a small proportion of correlations. We removed items with lower than 25% of correlations being significantly different at the 0.05 alpha level (Jones, 2024). To estimate the network, we used the Gaussian Graphical Model (GGM), in which edges correspond to partial correlation coefficients, thus representing the association between any two nodes after controlling for all other nodes in the network (Epskamp and Fried, 2018). GGM was estimated using EBICglasso. The EBICglasso algorithm applies the graphical least absolute shrinkage and selection operator (glasso) regularization (Friedman et al., 2008) combined with extended Bayesian information criterion (EBIC) model selection (Chen and Chen, 2008). This procedure sets trivial edges to zero to avoid false positives and provide a network with fewer edges. To maintain a balance between including true edges and excluding spurious ones, we set the hyperparameter *γ* at 0.5 (Foygel and Drton, 2010). A *γ* of 0 is less conservative potentially retaining false edges, while a *γ* of 1 is more conservative, potentially omitting true edges.

The accuracy of edge weights was examined using non-parametric bootstrapped 95% confidence intervals (CIs), with narrower CIs indicating more accurate edge weight estimates (Epskamp et al., 2018). Edge weights of 0.15 were considered small, 0.25 moderate, and 0.35 large (Christensen and Golino, 2021).

To assess the importance of each node in the network, we considered node strength, which is the sum of the absolute weights of all edges connected to the node (Opsahl et al., 2010). Higher values indicate a node’s greater importance to the network. The stability of strength centrality estimates was assessed by computing the correlation stability (CS) coefficient with case-dropping bootstrap, which examines whether the order of centrality estimates remains the same after estimating the network with less cases (Epskamp et al., 2018). The CS coefficient represents the maximum proportion of cases that can be dropped while maintaining a correlation of 0.70 between the centrality estimates of the original sample and those of the subsamples with a 95% probability. CS-coefficient values above 0.50 indicate adequate stability and interpretability (Epskamp et al., 2018).

To explore possible gender differences, men and women’s networks were compared via the Network Comparison Test (NCT) (van Borkulo et al., 2024). The NCT examines invariance of global network structure (i.e., whether the way nodes are connected is similar across groups), global network connectivity (i.e., whether the sum of the absolute values of all edge weights is similar across groups), and individual edge weights and node strength (i.e., whether a specific edge’s weight and a specific node’s strength is similar across groups, respectively). Analyses were conducted in R version 4.3.2 (R Core Team, 2023). We used the goldbricker function included in the package networktool (version 1.5.2) to identify redundant nodes (Jones, 2024), the package bootnet (version 1.5.3) (Epskamp et al., 2018) to estimate the networks and their accuracy and stability, and the package qgraph (version 1.9.8) (Epskamp et al., 2012) to plot networks and strength centrality. The package NetworkComparisonTest version 2.2.2 was used for analysis of network invariance across gender (van Borkulo et al., 2024).

## 3 Results

### 3.1 Sample characteristics

A total of 992 Italian adults meeting the inclusion criteria agreed to participate. Participants were 487 men (49.1%) and 505 women (50.9%), aged between 18 and 71 years (*M* = 36.40, SD = 13.75) and with a mean education of 15.35 years (SD = 3.31, range 8–22). About 75% of participants (*n* = 747) were employed, 57.8% (*n* = 573) were cohabiting with their partner, and 39.4% (*n* = 391) had children. Relationship length ranged between 1 and 52 years (*M* = 12.65 years, SD = 12.42). Women (*M* = 16.04, SD = 3.02) were significantly, moderately more educated than men (*M* = 14.64, SD = 3.44), *F*(1,990) = 46.51, *p* < 0.001, *d* = 0.43, and a slightly larger proportion of men (84.8%) than women (66.1%) were employed, *χ*^2^(1) = 46.45, *p* < 0.001, *V* = 0.22. Men and women did not differ in any other socio-demographic characteristics. As shown in Table 1, men scored significantly higher than women across all positive sexuality items and in self-rated physical health, with small effect sizes. Women were slightly more please than men with the personal characteristics and habits of their partner.

**Table 1:** Descriptive statistics (*M*, *SD*) of study variables by gender and effect size (Cohen’s *d*) for gender differences.

Node label	Item	Abbreviation	Men	Women	Cohen’s *d*
*Positive sexuality*					
PSS1	Sex brings a sense of fulfilment in my couple relationship.	Fulfilment	5.97 (1.30)	5.61 (1.51)	0.26^***^
PSS2	Sex with my partner is a beautiful experience.	Beautiful experience	6.28 (1.13)	6.05 (1.31)	0.19^**^
PSS3	Our intimate relationship is sexually stimulating.	Stimulating	6.04 (1.30)	5.67 (1.48)	0.27^***^
PSS4	Sex brings fun and joy in my couple relationship.	Fun	6.14 (1.31)	5.71 (1.55)	0.30^***^
PSS5	Sex with my partner is an exciting experience.	Exciting	6.14 (1.26)	5.72 (1.47)	0.31^***^
*Relationship satisfaction*					
EMS2	I am not pleased with the personality characteristics and personal habits of my partner.	Personality issues	3.88 (1.11)	4.05 (1.06)	0.16^*^
EMS3	I am very happy with how we handle role responsibilities in our relationship.	Equalitarian roles	4.30 (0.79)	4.21 (0.92)	0.10
EMS5	I am not happy about our communication and feel my partner does not understand me.	Communication	4.13 (1.04)	4.08 (1.07)	0.05
EMS7	I am very happy about how we make decisions and resolve conflicts.	Conflict resolution	4.05 (0.97)	4.01 (0.93)	0.04
EMS8	I am unhappy about our financial position and the way we make financial decisions.	Financial management	3.98 (1.21)	3.93 (1.18)	0.04
EMS10	I am very happy with how we manage our leisure activities and the time we spend together.	Leisure activities	4.02 (0.94)	3.98 (1.01)	0.04
EMS11	I am very pleased about how we express affection and relate sexually.	Sexual relationship	4.03 (1.02)	4.03 (0.99)	0.00
EMS14	I am dissatisfied about our relationship with my parents, in-laws, and/or friends.	Family and friends	4.05 (1.20)	4.00 (1.24)	0.04
EMS15	I feel very good about how we each practice our beliefs and values.	Beliefs and values	4.37 (0.80)	4.30 (0.83)	0.09
*Physical and mental health*					
PH	How would you define your physical health?	Physical health	7.74 (1.35)	7.52 (1.35)	0.16 ^*^
MH	How would you define your mental health?	Mental health	7.93 (1.46)	7.93 (1.40)	0.00

### 3.2 Item selection

The goldbricker function revealed one pair of overlapping EMS items (EMS3 and EMS7), which had 7.14% of statistically different correlations with other nodes. We therefore removed item EMS3, resulting in a total of 15 nodes included in the network.

### 3.3 Network structure

In the total sample (*n* = 992), 72 out of 105 possible edges (69%) were nonzero (mean edge weight 0.06, range 0.01–0.42), and all nodes were positively associated. Physical and mental health were connected by the largest edge weight (*r* = 0.42). There were moderate-to-large edges connecting PSS5 (exciting) with PSS2 (beautiful experience) (*r* = 0.32), PSS3 (stimulating) (*r* = 0.31), and PSS4 (fun) (*r* = 0.30), and between EMS2 (personality issues) and EMS5 (communication) (*r* = 0.30). A moderate edge connected PSS1 (fulfillent) and PSS4 (fun) (*r* = 0.26), and there were small-to-moderate edge weights between EMS10 (leisure activities) and EMS11 (sexual relationship) (*r* = 0.22), and between EMS5 (communication) and EMS7 (conflict resolution) (*r* = 0.21). Mean edge weight was 0.21 across PSS nodes, and 0.09 across EMS nodes.

**Figure 1:**
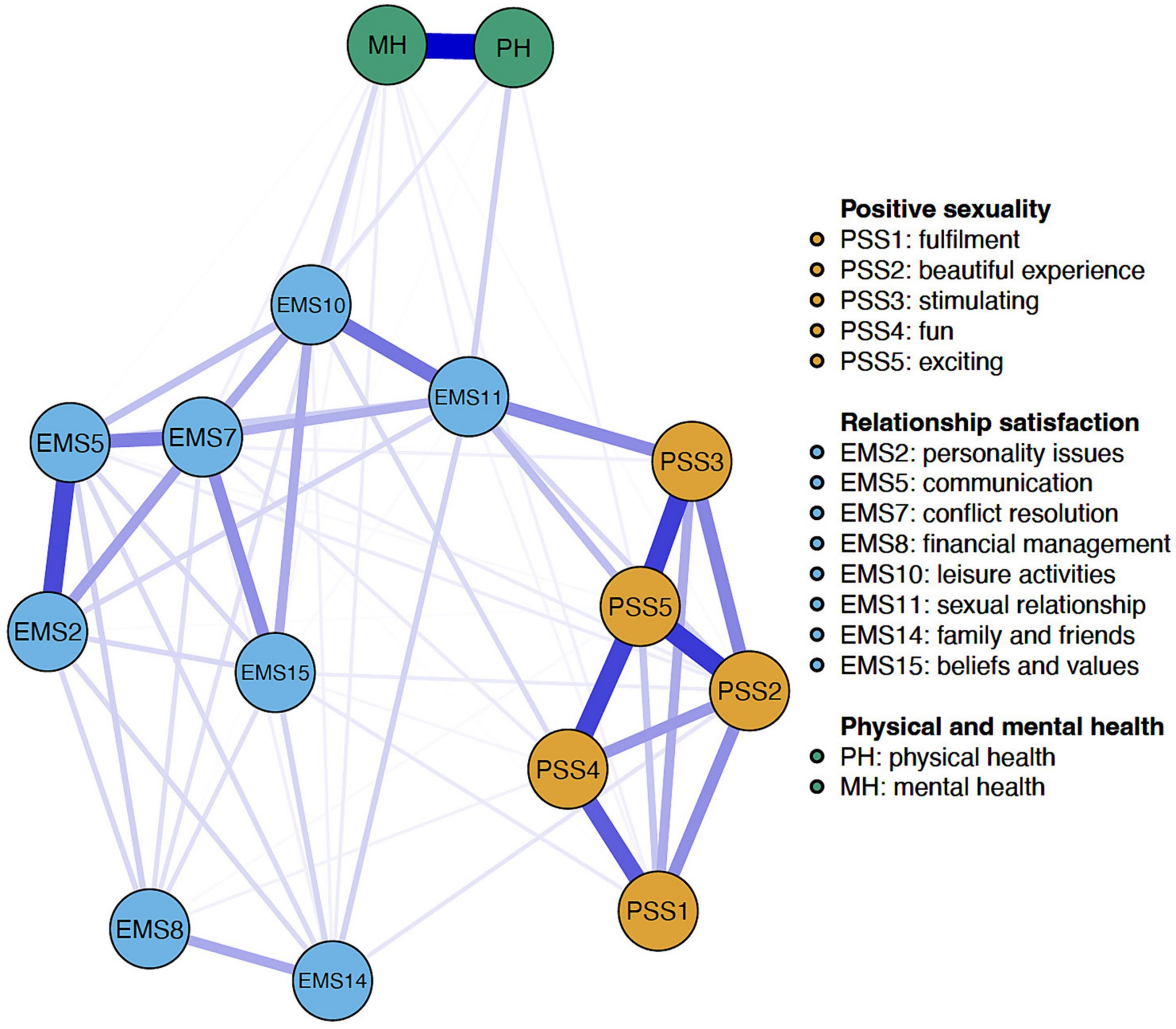
Network structure for the total sample. Blue edges indicate positive partial correlations, and red edges indicate negative partial correlations. Thicker and more saturated lines represent stronger connections.

The edges between PSS and EMS nodes were all negligible except the edge connecting EMS11 (sexual relationship) with PSS3 (stimulating) (*r* = 0.19) and PSS5 (exciting) (*r* = 0.11), which were small. Health nodes had negligible associations with both positive sexuality and relationship satisfaction nodes. The network structure for the total sample is depicted in Figure 1. Detailed information on edge weights is provided in Supplementary Table S1 in the Supplementary materials.

### 3.4 Node centrality

**Figure 2:**
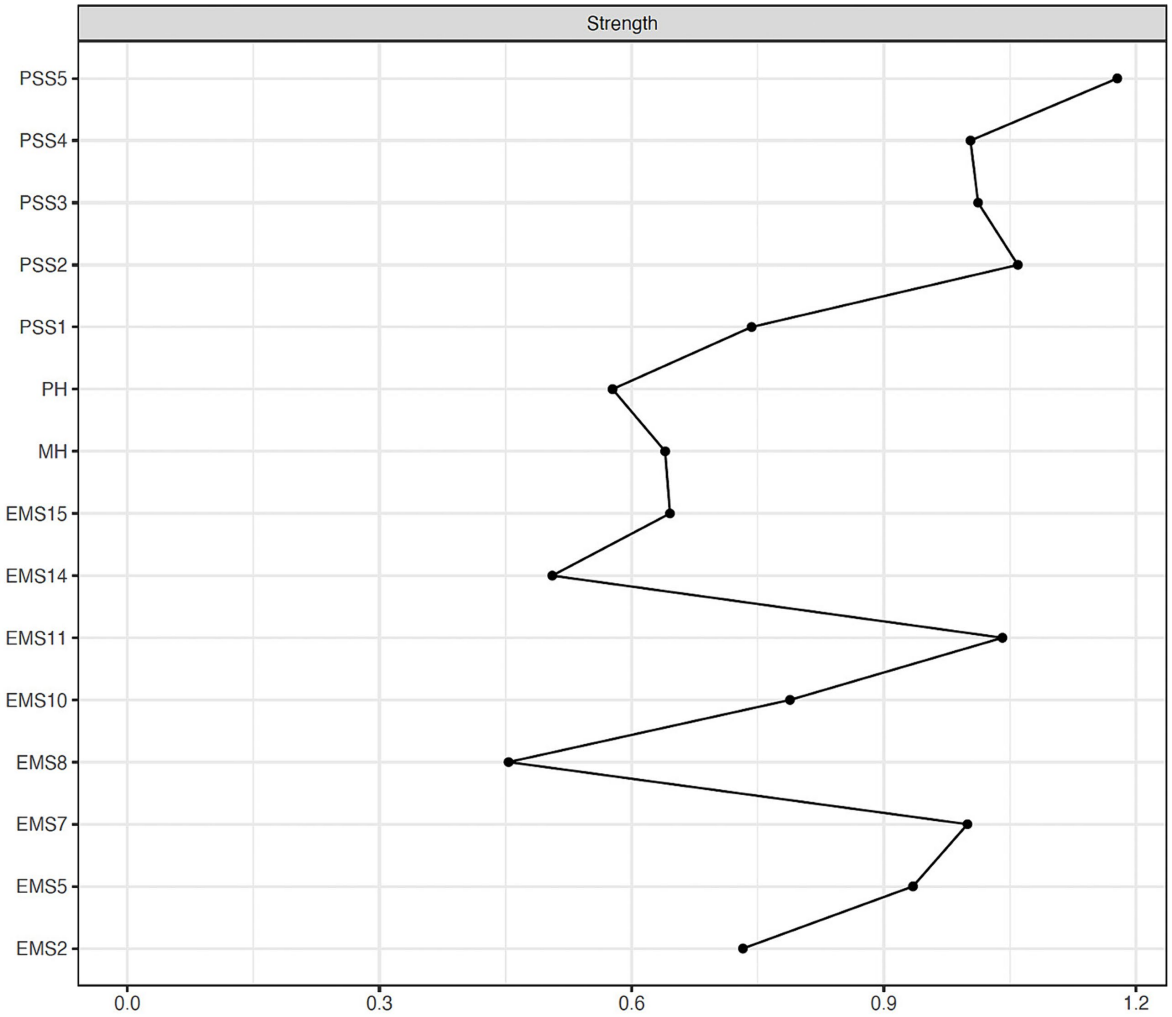
Node strength centrality estimates (unstandardized) for the total sample.

In the total sample, the node with the highest strength centrality was PSS5 (exciting) (1.18), followed by PSS2 (beautiful experience) (1.06) and EMS11 (sexual relationship) (1.04). The nodes with the lowest strength values were EMS14 (family and friends) (0.51) and EMS8 (financial management) (0.45). Centrality estimates are plotted in Figure 2. Exact node strength values are reported in Supplementary Table S2.

### 3.5 Network accuracy and stability

Results of accuracy and stability analysis indicated that the network was accurately estimated. As for network accuracy, the 95% CIs around edge weights that were obtained from 2,000 bootstrap samples were relatively narrow. In terms of network stability, the CS coefficient was 0.75, indicating adequate stability of strength centrality. The full results of accuracy and stability analyses are available in Supplementary Figures S1, S2, respectively.

### 3.6 Network comparison across gender

**Figure 3:**
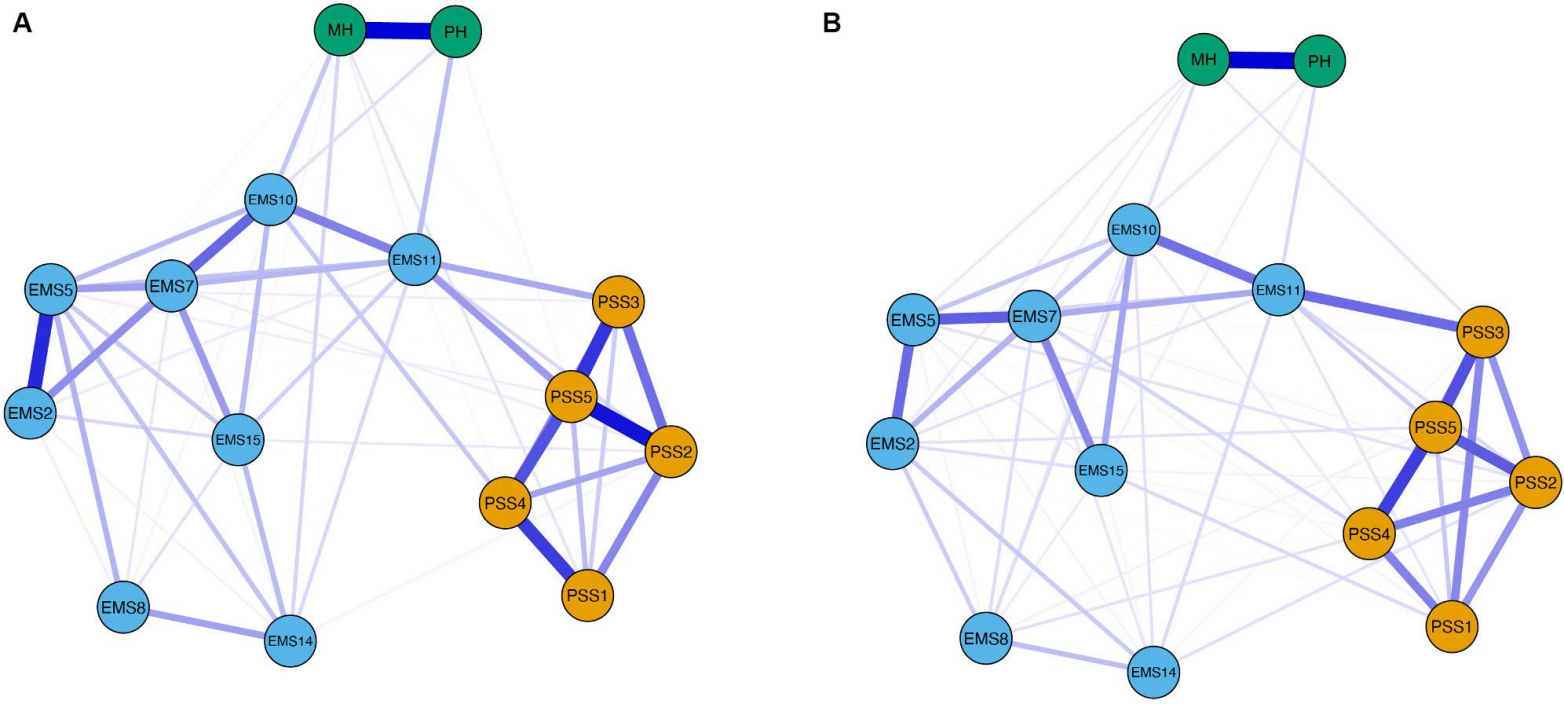
Network structure for men (*n* = 487) **(A)** and women (*n* = 505) **(B)**. Blue edges indicate positive partial correlations, and red edges indicate negative partial correlations. Thicker and more saturated lines represent stronger connections.

Of 105 possible edges, 60 (57%) and 75 (71%) had nonzero weights in men’s and women’s networks, respectively. In both networks, mean edge weight was 0.06 (range − 0.01-0.38 for men, 0.01–0.42 for women). Detailed edge weights by gender are reported in Supplementary Table S1. Men’s and women’s networks are displayed in Figure 3.

The NCT indicated that men’s and women’s networks did not differ in global network structure (*M* = 0.20, *p* = 0.066) and connectivity (*S* = 0.24, *p* = 0.126; *S_men_* = 5.95, *S_women_* = 6.19). Seven edge weights significantly differed across gender. The edge weight between EMS7 (conflict resolution) and EMS10 (leisure activities) was larger in men (0.23) than in women (0.03), *E* = 0.202, *p* = 0.003, while the edge between EMS11 (sexual relationship) and EMS15 (beliefs and values) was present in men’s (0.07) but not in women’s network, *E* = 0.070, *p* = 0.033. The edges connecting EMS2 (personality issues) with PSS5 (exciting) (women = 0.05; *E* = 0.046, *p* = 0.021) and EMS10 (leisure activities) (women = 0.11; *E* = 0.113, *p* = 0.023), and the edges between PSS4 (fun) and EMS7 (conflict resolution) (women = 0.07; *E* = 0.071, *p* = 0.041), and between PSS3 (stimulating) and MH (mental health) (women = 0.04; *E* = 0.042, *p* = 0.005) were present in women’s network only. The negligible edge weight between EMS2 (personality issues) and MH (mental health) had a positive sign in women (0.03) and a negative sign in men (−0.01), *E* = 0.039, *p* = 0.043.

**Figure 4:**
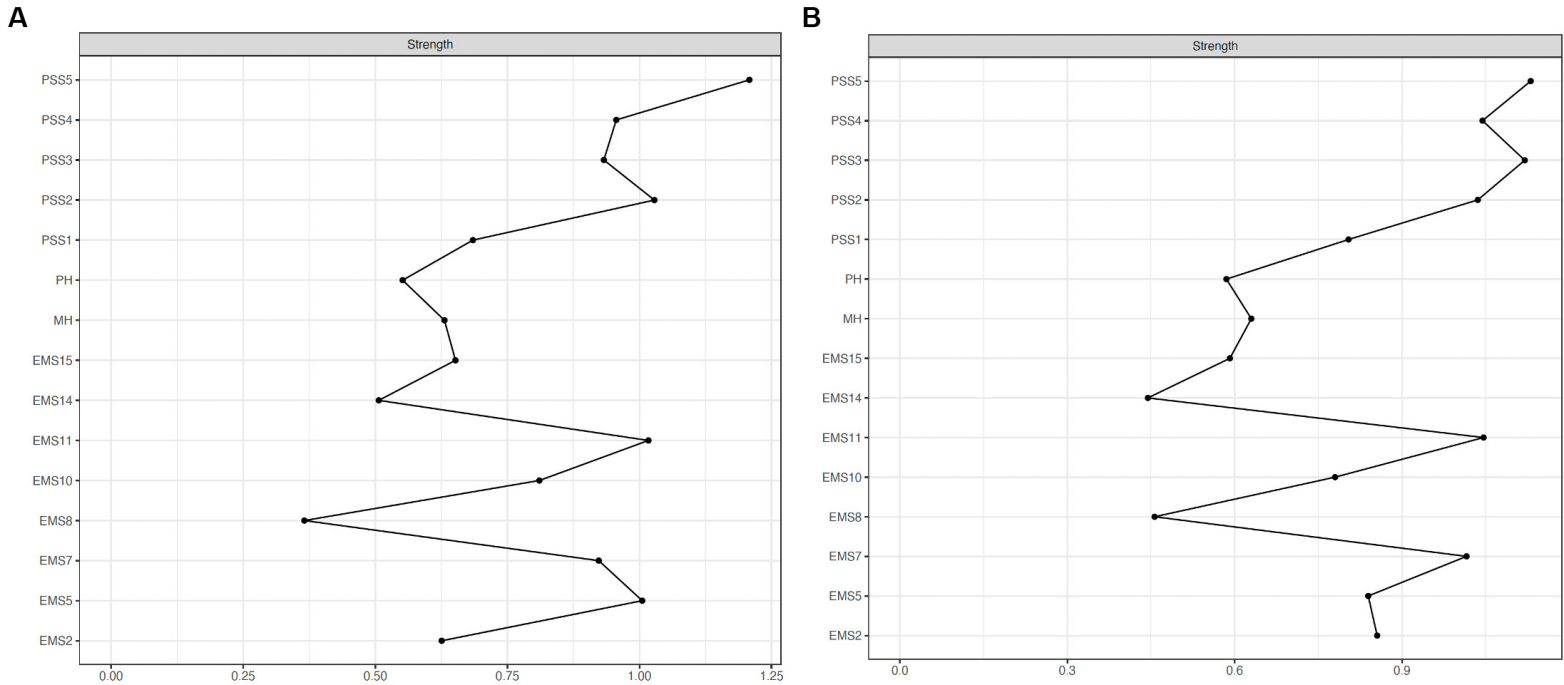
Node strength centrality estimates (unstandardized) for men **(A)** and women **(B)**.

Two node strengths significantly differed across gender. The strengths of nodes PSS3 (stimulating) (men = 0.93, women = 1.12; *p* = 0.027) and EMS2 (personality issues) (men = 0.63, women = 0.86; *p* = 0.031) were significantly higher in women than in men. Indeed, as shown in Figure 4, PSS3 (stimulating) was the second most central node in women’s network, while it was only the sixth most central node in men’s network. EMS2 (personality issues) was the eleventh and sixth most central node in men’s and women’s network, respectively. The remaining nodes had similar centrality levels across gender. For both men and women, the node with the highest strength centrality was PSS5 (exciting), and the nodes with the lowest centrality values were EMS8 (financial management) and EMS14 (family and friends). Strength centrality values by gender are available in Supplementary Table S2.

Based on accuracy and stability analysis, men’s and women’s networks were both accurately estimated, with relatively narrow 95% CIs around edge weights and a CS coefficient of 0.75 in both networks. Results of accuracy and stability analyses are presented in Supplementary Figures S3, S4, respectively.

## 4 Discussion

This was the first study, to our knowledge, to use network analysis to examine the associations between positive sexuality, relationship satisfaction and health variables, and explore potential gender differences in the network.

In the total sample, variables related to positive sexuality were more highly interconnected than the rest of the network. Notably, node PSS5 (“Sex with my partner is an exciting experience”) showed moderate-to-large associations with almost all other nodes in the same positive sexuality cluster. This is coherent with the sex-positive perspective, where sexual arousal and excitement are recognized as crucial components to understand and enhance sexual well-being (Werner et al., 2023; Williams et al., 2023). It also aligns with evidence that feeling sexually aroused within monogamous, heterosexual romantic relationships accounted for a considerable amount of the variance in sexual satisfaction for both men and women, as partner-induced sexual arousal may increase the amount of pleasure and enjoyment derived from sexual activity within the couple (Lawless et al., 2022). There was also a moderate connection between PSS1 (“Sex brings a sense of fulfillent in my couple relationship”) and PSS4 (“Sex brings fun and joy in my couple relationship”), underscoring that both the emotional and pleasurable aspects of sexual activity are interconnected and contribute to the quality of the couple’s relationship (Blumenstock, 2022).

Among relationship satisfaction nodes, EMS5 (“I am not happy about our communication and feel my partner does not understand me,” reverse coded) had a moderate-to-large connection with EMS2 (“I am not pleased with the personality characteristics and personal habits of my partner,” reverse coded), and a small-to-moderate association with EMS7 (“I am very happy about how we make decisions and resolve conflicts”). This is coherent with findings pointing to the positive impact of good communication on the perception of a partner’s behaviors and personal characteristics and on conflict resolution, which may ultimately enhance relationship satisfaction (Li et al., 2018; Pagani et al., 2019; Karakose and Ledermann, 2023). Noteworthy, communication and personality issues were connected by the same edge weight in a study of older couples that explored the network of marital satisfaction using the ENRICH marital satisfaction scale (Li et al., 2022). A small-to-moderate edge connected nodes EMS10 (“I am very happy with how we manage our leisure activities and the time we spend together”) and EMS11 (“I am very pleased about how we express affection and relate sexuality”). This association aligns with the recently proposed sex-as-leisure perspective, which emphasizes that positive sexuality, by connecting sexual behavior and expression to happiness and well-being, is consistent with leisure engagement (Berdychevsky and Carr, 2020; Williams et al., 2020). Previous research involving older couples (Li et al., 2022) found a stronger, moderate-to-large connection between nodes EMS10 and EMS11, although in that study, EMS11 exclusively addressed expression of affection, omitting any mention of sexuality. The difference in effect size might be attributable to the different item wording, as in later life stages, non-sexual forms of affection may become more significant (Towler et al., 2023), resulting in a stronger link with partnership facets such as shared activities.

Overall, the nodes related to positive sexuality, relationship satisfaction and health exhibited only small or negligible associations with each other. Two small connections were observed between PSS and EMS variables. The links of node EMS11 (“I am very pleased about how we express affection and relate sexually”) with nodes PSS3 (“Our intimate relationship is sexually stimulating”) and PSS5 (“Sex with my partner is an exciting experience”) align with evidence that communication allowing partners to express their emotions, desires and challenges regarding sexuality helps maintaining sexual passion and desire within the relationship (Frederick et al., 2017; Mallory, 2022). However, it should be noted that item EMS11, despite belonging to the relationship satisfaction measure used in this study, refers to sexual relationship (Fowers and Olson, 1993), which likely accounts for its link with positive sexuality. The associations of health variables with both positive sexuality and relationship satisfaction were either negligible or non-existent. This finding contrasts previous evidence emphasizing the connections of health with positive aspects of sexuality (Flynn et al., 2016; Casu, 2019) and relationship satisfaction (Farrell and Simpson, 2017; Mónaco et al., 2021). Nonetheless, as suggested by other research, these links may be indirect and mediated by factors such as perceived stress (Wei et al., 2024) and body image (Spivak-Lavi and Gewirtz-Meydan, 2022), among others. Notably, the strongest association within the network was between mental and physical health. While coherent with the well-established, strong link between psychological well-being and physical health (e.g., Kesavayuth et al., 2022; Yang and Zikos, 2022), this result might be attributable to the fact that health nodes were the only intrapersonal factors considered in the network, while PSS and EMS nodes were all inherently dyadic.

Based on strength centrality values, the most important variables in the total sample were PSS5 (“Sex with my partner is an exciting experience”), PSS2 (“Sex with my partner is a beautiful experience”), and EMS11 (“I am very pleased about how we express affection and relate sexually”), which had the highest number and magnitude of direct connections with other nodes. This is in line with findings from an exploratory network analysis of sexual and relationship satisfaction among partnered individuals, in which sexual pleasure, desire, and satisfaction were among the most central nodes (Nickull et al., 2022). Node EMS8 (“I am unhappy about our financial position and the way we make financial decisions”) was the least important in the network, consistent with findings from the original validation study of the ENRICH Marital Satisfaction Scale, where this item had the lowest item-total correlation (Fowers and Olson, 1993). The observed prominence of sexuality-related nodes in the present study suggests that interventions targeting relationship issues should focus on enhancing the experience of sexual life with the partner as beautiful and exciting as the initial area of focus.

In comparing men’s and women’s networks, the NCT showed no significant differences in overall network structure and connectivity, consistent with previous network analyses on the connections among positive sexuality, relationship satisfaction, and/or health variables across genders (Gustavson et al., 2016; Carcedo et al., 2020; Stephenson et al., 2021). However, a few gender differences were found at the local level. The positive connection between EMS7 (“I am very happy about how we make decisions and resolve conflicts”) and EMS10 (“I am very happy with how we manage our leisure activities and the time we spend together”) was moderate in men and negligible in women. On the other hand, only in women’s network, EMS7 was positively associated with PSS4 (“Sex brings fun and joy in my couple relationship”), although very weakly. This seems coherent with research indicating that men and women may value different aspects of relationship dynamics, probably due societal expectations and cultural narratives about masculinity and femininity (Ellemers, 2018; Cionea et al., 2019). Indeed, men are socialized to adopt a more problem-solving orientation within relationships (Brems and Johnson, 1989; Ptacek et al., 1992); therefore, successful decision making and conflict resolution might be a fundamental component of the enjoyment they derive from leisure time spent with their partners. Differently, women are generally socialized to place greater emphasis on emotional connection and intimacy in close relationships (Horne and Johnson, 2018); therefore, successfully solving conflicts within the couple might contribute to women’s sexual fulfillent by deepening emotional closeness. However, a positive association between EMS11 (“I am very pleased about how we express affection and relate sexually”) and EMS15 (“I feel very good about how we each practice our beliefs and values”) was present in men’s but not in women’s network. This contrasts with evidence suggesting that nonsexual, cognitive intimacy with the partner is a stronger driver of sexual satisfaction for women than for men (Rausch and Rettenberger, 2021; Józefacka et al., 2023). Considering that the effect size of this gender-specific association was negligible, further research is needed to clarify this finding.

Among women only, EMS2 (“I am not pleased with the personality characteristics and personal habits of my partner,” reverse coded) was positively associated with both PSS5 (“Sex with my partner is an exciting experience”) and EMS10 (“I am very happy with how we manage our leisure activities and the time we spend together”). This suggests that engaging in enjoyable shared activities may reinforce women’s positive views of their partners’ characteristics. In support of this suggestion, previous longitudinal findings indicated that positive, intimacy-related relationship experiences can promote a more favorable perception of close others and bolster relationship satisfaction and well-being (Stanton et al., 2017). However, some caution is warranted in interpreting these gender specific associations due to the very small effect size.

Finally, the edge connecting PSS3 (“Our intimate relationship is sexually stimulating”) and MH (self-rated mental health) was nonzero and positive only for women, albeit with a trivial effect size. This result fits the responsive model of sexual desire (Basson, 2000), which posits that a woman’s sexual desire is more reactive than spontaneous and is triggered in contexts that facilitate such desire, including her mental health status (Krasnow and Maglio, 2021).

Regarding gender differences in node strengths, node PSS3 (stimulating) was the second most central node for women, and the sixth most central node for men, indicating that women place great emphasis on how stimulating the sexual component of their intimate relationship is. Similarly, another network analysis exploring sexual and relationship satisfaction found that sexual desire and pleasurable partnered sex were more central in women’s than in men’s network (Nickull et al., 2022). Node EMS2 (personality issues) was the sixth most central node in women’s and the eleventh most central node in men’s network. Although this variable did not play a prominent role in either network, women attributed greater importance to their level of contentment with the partner’s personality characteristics, compared to men. The parental investment theory (Trivers, 1972) posits that women, investing more in offspring than men, are more selective in choosing a mate. Thus, they tend to prefer partners whose characteristics are indicative of suitability for long-term relationships, such as altruism or cooperativeness (Bhogal et al., 2019). Notably, as found in the total sample network, for both men and women the most central node was PSS5 (exciting), meaning that, regardless of gender, the perception of excitement and enjoyment in sexual encounters with the partner should be a primary aspect to be promoted in intimate relationships.

The present study has some limitations that must be acknowledged. First, the cross-sectional nature of our study prevents any causal inferences among positive sexuality, relationship satisfaction, and health variables. Future studies are warranted to clarify temporal relationships through prospective designs, such as intensive repeated measures with ecological momentary assessment (Wen et al., 2022; Pawłowska et al., 2023). Related to this, identifying central nodes within the networks as potential intervention targets could be improved by establishing their causal relationships with other symptoms (Borsboom et al., 2021). Second, only self-report measures were used in this study, which might result in artificially inflated shared variance between variables, and in motivationally biased perceptions of the partner and the relationship (Faure et al., 2024). Future studies should thus consider incorporating implicit and objective measures of relationship aspects, like sexual desire and communication patterns, in addition to self-reports (Righetti et al., 2022; Faure et al., 2024). Third, this study used two single-item measures for physical and mental health, thereby encompassing a more limited set of indicators and resulting in fewer nodes in the network compared to those for positive sexuality and relationship satisfaction. Future studies using network analysis should aim to include a wider array of variables relevant to both physical and mental health, as to elucidate their associations with positive sexuality and relationship quality. Also, variables that could impact aspects of sexual and relationship functioning, like sociosexual orientations, the internalization of gender stereotypes, and sexual coping mechanisms (Alvarado et al., 2020; Berdychevsky, 2022; Mallory, 2022) were not considered in this study and should be taken into account in future research. Fourth, we focused on positive sexuality defined as the positive feelings and attribution of positive meaning to one’s sexual experiences within a romantic couple. However, positive sexuality comprises a wider set of components, including attitudes toward sex, sexual fantasy, and sexual interests and behaviors (Williams et al., 2015), which deserve consideration in future research. Fifth, we did not correct for multiple testing when evaluating gender invariance of edge weights and node strengths in the NCT. Nonetheless, it has been argued that correcting for multiple testing in the NCT is not necessary when the analysis is exploratory, as is the case of gender comparisons in this study (van Borkulo et al., 2023). Lastly, this study was limited to men and women in heterosexual relationships from the Italian general population, thereby restricting the generalizability of our findings to binary gender identities within the same cultural context. To enhance our understanding of the links between positive sexuality, relationship satisfaction and health, future research should include diverse samples, such as non-binary individuals, those in same-sex relationships, those seeking therapy, and people from different cultural backgrounds. Another threat to the generalizability of our findings is our recruitment strategy. Our reliance on snowball sampling, which draws from personal networks and referrals, may have inadvertently resulted in the selection bias toward subgroups with similar traits or characteristics. Although costly, a random-sampling strategy would ideally minimize this risk and should be considered for future research.

Despite the above limitations, the findings of the present study have implications for both research and clinical practice. By highlighting the importance of pleasure in sexual experiences within couples, our study contributes to the WHO’s paradigm shift toward a more holistic and positive conceptualization of sexual health as a fundamental component of overall well-being. For positive sexuality research, this study provides a foundation for future exploration of the role of positive sexuality in personal and relational functioning. Sex-positive researchers are encouraged to more thoroughly explore how positive sexuality in intimate relationships relates to health and well-being, considering other aspects of physical and mental health and employing more detailed measurements. Additionally, a variety of relational variables relevant to positive sexuality, such as sexual communication, sexual practices and gender roles, should be examined to enhance our understanding of the dynamics between sexual and relationship functioning. In pursuing this, researchers are invited to adopt the network approach. Network analysis offers a unique data-driven method for investigating complex relationships between variables, enabling the identification of key variables and the discovery of unexpected connections (Hevey, 2018; Borsboom et al., 2021). Regarding clinical implications, our findings highlighted the prominent role of pleasurable feelings and sensations during partnered sex for both men and women. Therefore, couple and sex therapists/counselors should prioritize these aspects by implementing interventions aimed at amplifying positive feelings during partnered sexual intercourse. Strategies proven effective in improving erotic connection and desirable sex include recommending the use of sex toys and exercises that focus on paying attention to pleasurable sensations while touching and being touched (De Santis et al., 2019; Kleinplatz et al., 2020). Also, interventions aimed at enhancing communication skills within the couple (e.g., Rosier and Tyler, 2017; Morgis et al., 2019; Leavitt et al., 2021) can assist clients in verbalizing and expressing their sexual needs and preferences to actively pursue pleasurable sexual encounters. Facilitating honest discussions between partners about their values and aspirations related to relationships and sexuality, including erotic preferences and fantasies, may encourage the recognition and acceptance of one’s right to pleasure and enhance the enjoyment derived from sexual interactions (Nimbi et al., 2021; Mallory, 2022). Moreover, to effectively promote sexual health, well-being, and enjoyment, practitioners are encouraged to adopt a more holistic, rights-based, and positive framework in sexuality education. This approach should include efforts to enhance sexual knowledge, encourage communication about sex, foster sexual self-efficacy, and promote a positive and inclusive orientation toward pleasure (McFarland and Williams, 2016; Nimbi et al., 2021; Mollen and Abbott, 2022). In particular, marriage counselors and relationship experts should consider incorporating sex-positive education interventions focused on sex as leisure activity, which have been proposed to empower individuals to take control over their sex lives, thus promoting sexual agency and well-being (Berdychevsky, 2022).

Regarding the implications for the average person, this study underscores the significance of pleasurable experiences during partnered sexual intercourse to enhance the overall quality of a relationship. Couples might benefit from prioritizing sexual communication and normalizing the discussion of sexual preferences and desires, which has been shown to promote sexual and relationship satisfaction (Jones et al., 2018). In particular, women, who in this study placed higher value on how sexually stimulating their intimate relationships are, might consider taking an assertive role in creating enjoyable shared activities during sex. This can be achieved by communicating effectively about what they like and want during sexual activities (Dastyar et al., 2018). By focusing on these aspects, both partners can work together to make their relationship more satisfying and fulfilling. Altogether, increased awareness among relationship experts and the general population about actions that can foster positive sexuality may ultimately contribute to the World Health Organization’s view of sexual health as encompassing more than just the absence of disease and the presence of sexual function.

In conclusion, we found small-to-negligible connections between positive sexuality and relationship satisfaction, both of which had negligible or no connections with health. The interconnections among positive sexuality variables highlighted the importance of sexual arousal and excitement, and the contribution of both emotional and pleasurable aspects of sexual intercourse to the overall quality of the relationship. Good partner communication emerged as relevant for relationship satisfaction, and both sexual and non-sexual forms of affection were linked to sexual arousal and excitement during partnered sex. The network was globally invariant across gender, although a few connections were gender-specific. Regardless of gender, the most important variables in the network were related to pleasurable feelings and sensations during sexual intercourse with the partner. This study contributes to the academic discourse on positive sexuality and offers practical insights for improving intimate relationships. However, future research should consider more comprehensive models to further explore the intricate interplay between positive sexuality dimensions and personal and relational outcomes.

## Data availability statement

The datasets presented in this study can be found in online repositories. The names of the repository/repositories and accession number(s) can be found at: https://osf.io/75cyr/.

## Ethics statement

The studies involving humans were approved by the Bioethics Committee of University of Bologna. The studies were conducted in accordance with the local legislation and institutional requirements. The participants provided their written informed consent to participate in this study.

## Author contributions

GA: Formal analysis, Writing – original draft. CR: Methodology, Supervision, Writing – review & editing. PG: Data curation, Investigation, Methodology, Supervision, Writing – review & editing. SH: Methodology, Supervision, Writing – review & editing. GC: Conceptualization, Data curation, Formal analysis, Investigation, Supervision, Writing – original draft, Writing – review & editing.
