# Supplementary material for: Positive sexuality, relationship satisfaction, and health: a network analysis
Source: Front Psychol. 2024 Jun 6;15:1420148. doi: 10.3389/fpsyg.2024.1420148 (PMC11189356; doi:10.3389/fpsyg.2024.1420148)
Supplement: Supplementary file 1 [file Data_Sheet_1.pdf]

Supplementary Material

Table S1. Edge weights in the total sample and separately by gender.

**Total sample (*n* = 992)**														
	**PSS1**	**PSS2**	**PSS3**	**PSS4**	**PSS5**	**EMS2**	**EMS5**	**EMS7**	**EMS8**	**EMS10**	**EMS11**	**EMS14**	**EMS15**	**PH**
**PSS2**	0.180													
**PSS3**	0.136	0.197												
**PSS4**	0.262	0.165	0.152											
**PSS5**	0.092	0.320	0.313	0.304										
**EMS2**	0	0	0	0	0.005									
**EMS5**	0	0.028	0.024	0	0.008	0.295								
**EMS7**	0.003	0.028	0.003	0.030	0	0.151	0.206							
**EMS8**	0	0	0	0.020	0.007	0.062	0.075	0.051						
**EMS10**	0	0	0	0.056	0	0.035	0.101	0.134	0					
**EMS11**	0.015	0.064	0.188	0	0.106	0.063	0.077	0.128	0	0.223				
**EMS14**	0	0.039	0	0	0	0.059	0.057	0.024	0.139	0.021	0.071			
**EMS15**	0.032	0.030	0	0.013	0	0.061	0.060	0.179	0.048	0.135	0.007	0.067		
**PH**	0	0	0	0	0.021	0	0	0.040	0.003	0.023	0.075	0	0	
**MH**	0.022	0.007	0	0	0	0	0.003	0.023	0.048	0.058	0.023	0.028	0.013	0.415
**Men (*n* = 487)**														
	**PSS1**	**PSS2**	**PSS3**	**PSS4**	**PSS5**	**EMS2**	**EMS5**	**EMS7**	**EMS8**	**EMS10**	**EMS11**	**EMS14**	**EMS15**	**PH**
**PSS2**	0.179													
**PSS3**	0.077	0.216												
**PSS4**	0.293	0.135	0.176											
**PSS5**	0.099	0.356	0.305	0.260										
**EMS2**	0	0	0.003	0	0									
**EMS5**	0	0.004	0.025	0	0.025	0.322								
**EMS7**	0	0.025	0	0	0	0.167	0.146							
**EMS8**	0	0	0	0	0	0.021	0.114	0.034						
**EMS10**	0	0.004	0	0.082	0	0	0.105	0.229	0					
**EMS11**	0	0.057	0.130	0.010	0.151	0.028	0.092	0.109	0	0.190				

**EMS14**	0	0.016	0	0	0	0.018	0.091	0.016	0.140	0	0.059			
**EMS15**	0	0.032	0	0	0	0.058	0.082	0.150	0.041	0.106	0.070	0.103		
**PH**	0	0	0	0	0.012	0	0	0.046	0	0.009	0.101	0	0	
**MH**	0.037	0.005	0	0	0	-0.009	0	0	0.015	0.086	0.021	0.063	0.010	0.383
**Women (*n* = 505)**														
	**PSS1**	**PSS2**	**PSS3**	**PSS4**	**PSS5**	**EMS2**	**EMS5**	**EMS7**	**EMS8**	**EMS10**	**EMS11**	**EMS14**	**EMS15**	**PH**
**PSS2**	0.178													
**PSS3**	0.198	0.180												
**PSS4**	0.209	0.211	0.130											
**PSS5**	0.092	0.271	0.292	0.322										
**EMS2**	0.007	0	0	0	0.046									
**EMS5**	0	0.048	0.007	0.009	0	0.250								
**EMS7**	0.017	0.018	0.005	0.071	0.002	0.125	0.268							
**EMS8**	0	0	0.002	0.047	0.021	0.084	0.017	0.057						
**EMS10**	0	0	0	0.032	0	0.113	0.096	0.027	0.031					
**EMS11**	0.041	0.062	0.249	0	0.081	0.043	0.065	0.143	0	0.239				
**EMS14**	0	0.050	0.014	0	0.002	0.088	0.018	0.040	0.109	0.047	0.061			
**EMS15**	0.061	0.019	0	0.014	0	0.054	0.027	0.197	0.030	0.149	0	0.015		
**PH**	0	0	0	0	0.000	0.015	0.001	0.020	0	0.033	0.063	0	0.025	
**MH**	0	0	0.042	0	0	0.030	0.034	0.026	0.057	0.013	0	0	0	0.427

PSS = Positive Sexuality Scale; EMS = ENRICH Marital Satisfaction Scale; PH = Physical Health; MH = Mental Health; PSS1 = Sex brings a sense of fulfilment in my couple relationship; PSS2 = Sex with my partner is a beautiful experience; PSS3 = Our intimate relationship is sexually stimulating; PSS4 = Sex brings fun and joy in my couple relationship; PSS5 = Sex with my partner is an exciting experience; EMS2 = I am not pleased with the personality characteristics and personal habits of my partner; EMS5 = I am not happy about our communication and feel my partner does not understand me; EMS7 = I am very happy about how we make decisions and resolve conflicts; EMS8 = I am unhappy about our financial position and the way we make financial decisions; EMS10 = I am very happy with how we manage our leisure activities and the time we spend together; EMS11 = I am very pleased about how we express affection and relate sexually; EMS14 = I am dissatisfied about our relationship with my parents, in-laws, and/or friends; EMS15 = I feel very good about how we each practice our beliefs and values; PH = How would you define your physical health?; MH = How would you define your mental health?

Table S2. Node strength values (strength rank) in the total sample and separately by gender.

Node label	Total sample	Men	Women
PSS1	0.74 (8)	0.68 (9)	0.80 (8)
PSS2	1.06 (2)	1.03 (2)	1.04 (4)
PSS3	1.01 (4)	0.93 (6)	1.12 (2)
PSS4	1.00 (5)	0.96 (5)	1.04 (4)
PSS5	1.18 (1)	1.21 (1)	1.13 (1)
EMS2	0.73 (9)	0.63 (11)	0.86 (6)
EMS5	0.93 (6)	1.01 (4)	0.84 (7)
EMS7	1.00 (5)	0.92 (7)	1.02 (5)
EMS8	0.45 (14)	0.37 (14)	0.46 (12)
EMS10	0.79 (7)	0.81 (8)	0.78 (9)
EMS11	1.04 (3)	1.02 (3)	1.05 (3)
EMS14	0.51 (13)	0.51 (13)	0.44 (13)
EMS15	0.65 (10)	0.65 (10)	0.59 (11)
PH	0.58 (12)	0.55 (12)	0.59 (11)
MH	0.64 (11)	0.63 (11)	0.63 (10)

PSS = Positive Sexuality Scale; EMS = ENRICH Marital Satisfaction Scale; PH = Physical Health; MH = Mental Health; PSS1 = Sex brings a sense of fulfilment in my couple relationship; PSS2 = Sex with my partner is a beautiful experience; PSS3 = Our intimate relationship is sexually stimulating; PSS4 = Sex brings fun and joy in my couple relationship; PSS5 = Sex with my partner is an exciting experience; EMS2 = I am not pleased with the personality characteristics and personal habits of my partner; EMS3 = I am very happy with how we handle role responsibilities in our relationship; EMS5 = I am not happy about our communication and feel my partner does not understand me; EMS7 = I am very happy about how we make decisions and resolve conflicts; EMS8 = I am unhappy about our financial position and the way we make financial decisions; EMS10 = I am very happy with how we manage our leisure activities and the time we spend together; EMS11 = I am very pleased about how we express affection and relate sexually; EMS14 = I am dissatisfied about our relationship with my parents, in-laws, and/or friends; EMS15 = I feel very good about how we each practice our beliefs and values; PH = How would you define your physical health?; MH = How would you define your mental health?

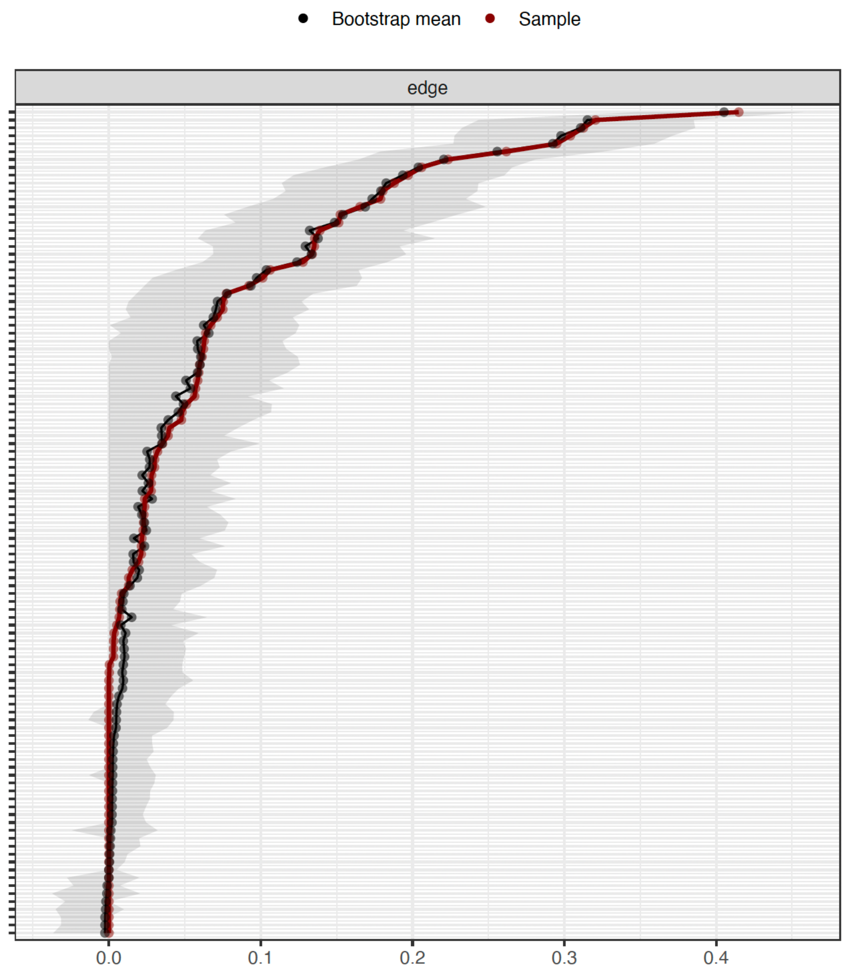

Figure S1. Accuracy of edge weights in the total sample. The red line shows the edge value estimated in the sample, the black line represents the average bootstrapped estimated edge value, and the gray area indicates the bootstrapped 95% CIs. Each horizontal line represents an edge, ordered from the highest to the lowest edge weight. Node labels on the Y-axis are omitted for clarity.

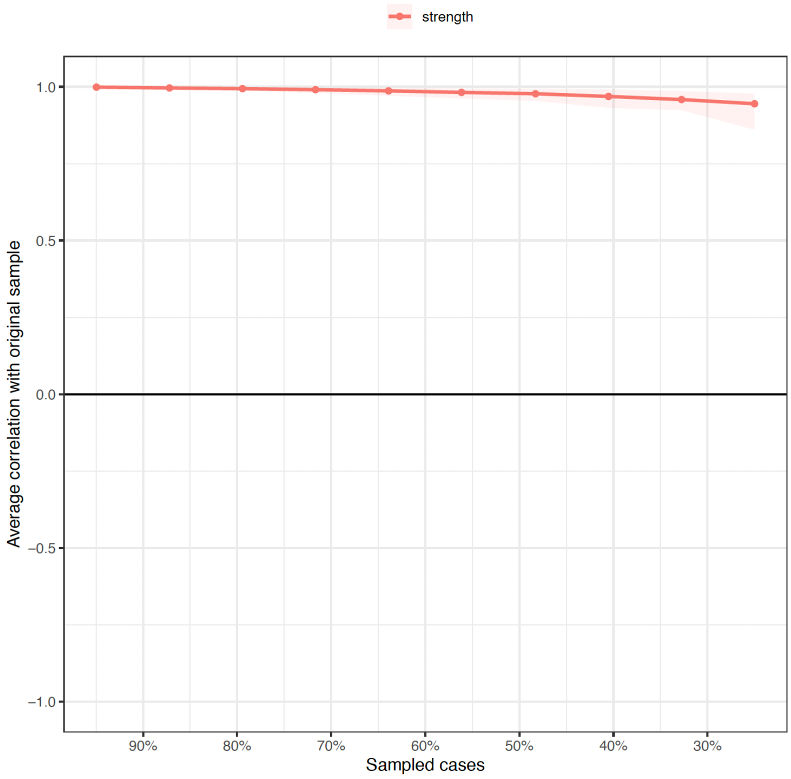

Figure S2. Stability of node strength centrality in the total sample. The red line represents the average correlation between strength centrality indices estimated in the original total sample and centrality indices obtained from case-dropping bootstrap. The area around the line represents the range of correlations from the 2.5^{th} quantile to the 97.5^{th} quantile.

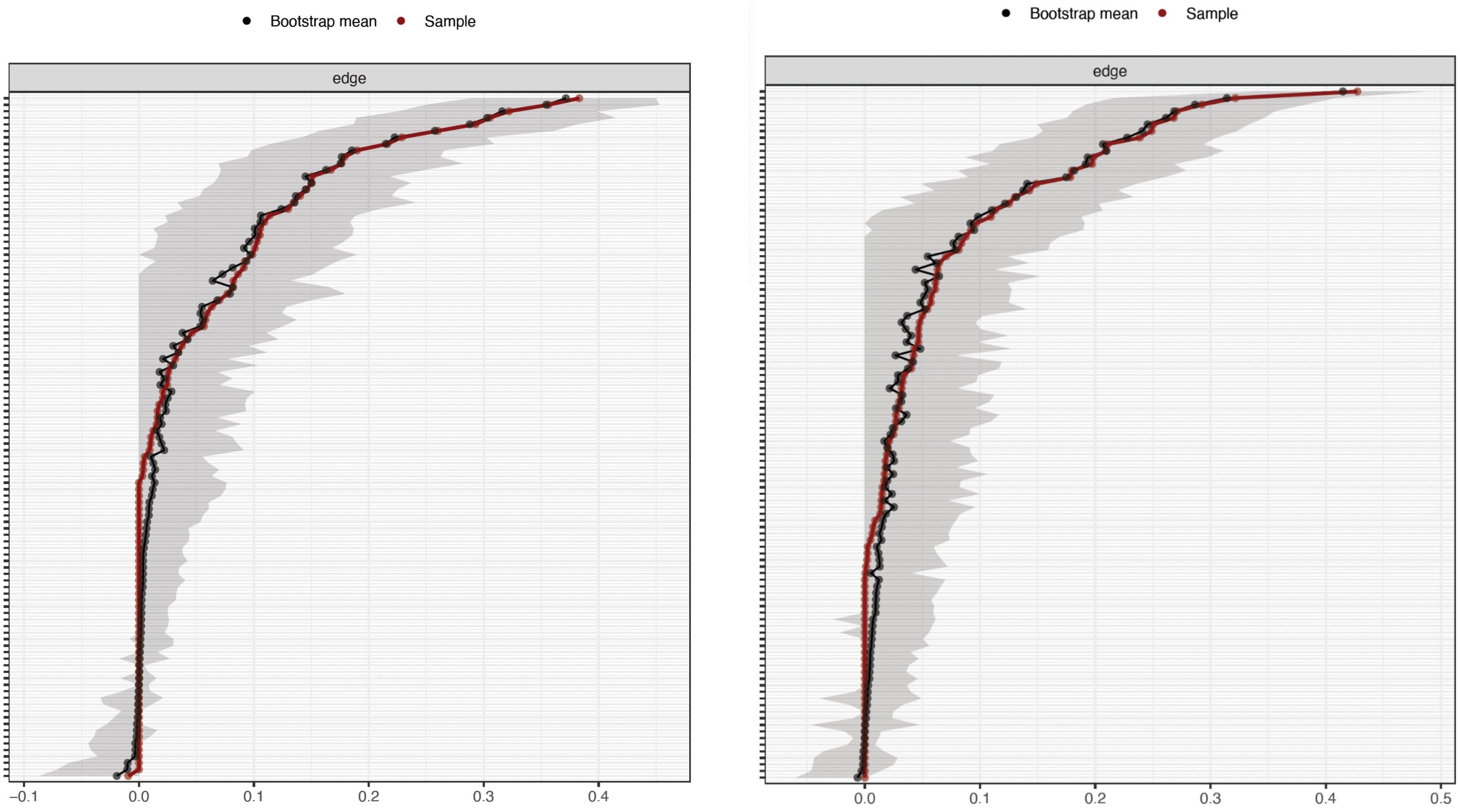

(A) **(B)**

Figure S3. Accuracy of edge weights for men **(A)** and women **(B)**. The red line shows the edge value estimated in the subsample, the black line represents the average bootstrapped estimated edge value, and the gray area indicates the bootstrapped 95% CIs. Each horizontal line represents an edge, ordered from the highest to the lowest edge weight. Node labels on the Y-axis are omitted for clarity.

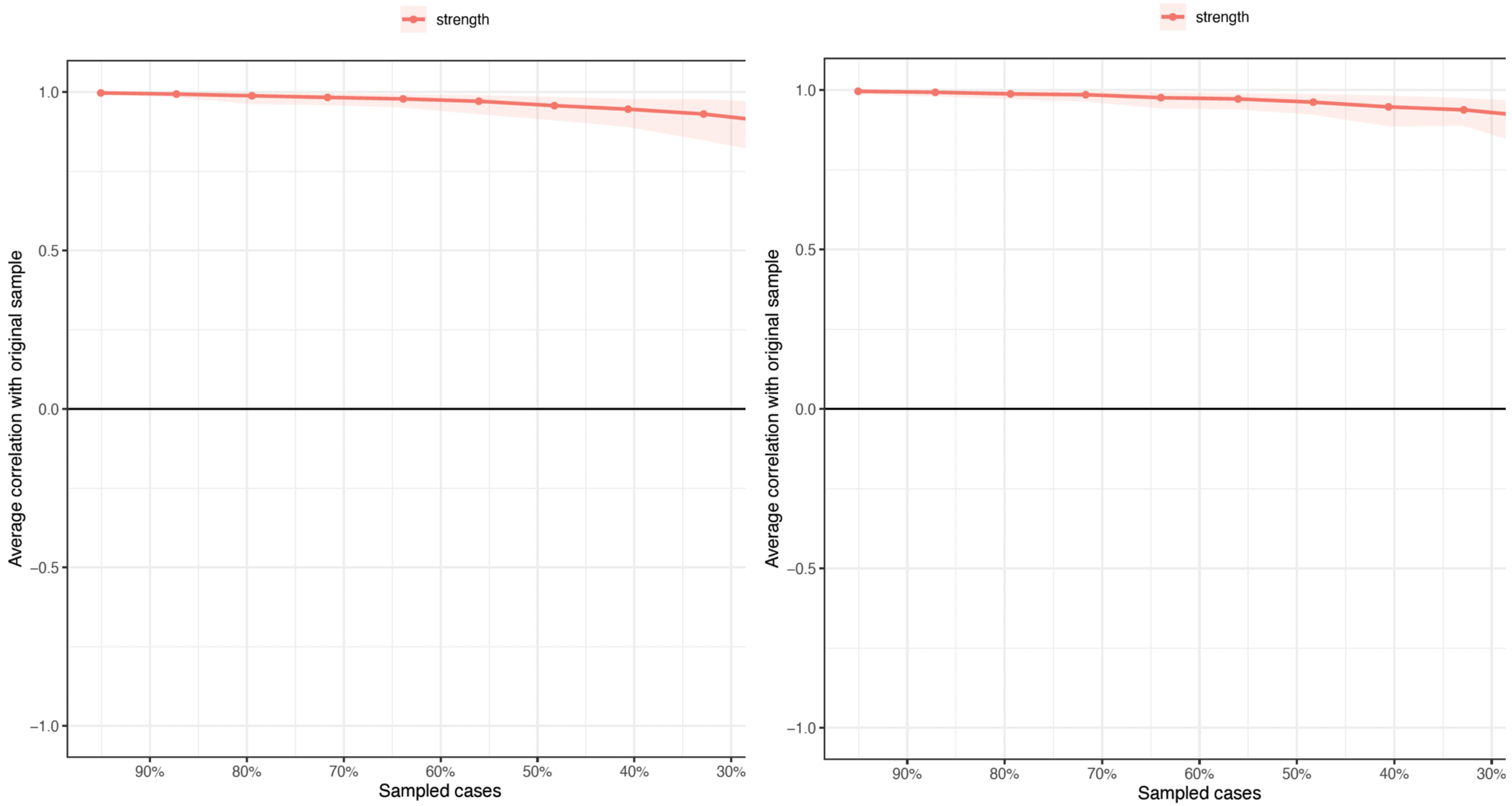

(A) **(B)**

Figure S4. "Stability of node strength centrality for men (**A**) and women (**B**). The red line represents the average correlation between strength centrality indices estimated in the original subsample and centrality indices obtained from case-dropping bootstrap. The area around the line represents the range of correlations from the 2.5th quantile to the 97.5th quantile.
